# Manifestations of Indirect Self-destructiveness and Methods of Suicide Attempts

**DOI:** 10.1007/s11126-012-9239-x

**Published:** 2012-10-04

**Authors:** Konstantinos Tsirigotis, Wojciech Gruszczynski, Marta Lewik-Tsirigotis

**Affiliations:** 1 Department of Psychology, The Jan Kochanowski University in Kielce, Piotrkow Trybunalski Branch, Słowackiego 114/118, 97-300 Piotrków Trybunalski, Poland; 2 Neurotic and Stress Related Disorders Clinic, The Medical University of Lodz, Lodz, Poland; 3 Division of Medicinal Chemistry and Microbiology, Chemistry Department, Wroclaw University of Technology, Wroclaw, Poland

**Keywords:** Indirect self-destructiveness, Transgression and Risk, Poor health maintenance, Personal and social neglects, Lack of planfulness, Helplessness and passiveness, Suicide attempt methods

## Abstract

The method of suicide attempt is related to motivational processes and the psycho(patho)logical mechanisms and traits of an individual. Indirect self-destructiveness is related to direct self-destructiveness. It is presumed that it can transform to the latter thus leading to suicide attempts or death by suicide. The study objective was to examine the relationship between individual manifestations of indirect self-destructiveness and the methods of suicide attempt as well as to explore the indirect predictors of particular suicide methods. The study was conducted among 147 persons (114 females, 33 males) who attempted suicide. The research instrument was the Polish version of the “Chronic Self-Destructiveness Scale” (CS-DS), including Transgression and Risk, Poor Health Maintenance, Personal and Social Neglects, Lack of Planfulness, and Helplessness and Passiveness in the face of problems. Correlation and regression analyses were applied. A number of statistically significant correlations were found between indirect self-destructiveness, or its manifestations, and the methods of suicide attempt. Moreover, the particular categories of indirect self-destructive behaviour were found to largely determine the choice of the method of suicide attempt. Among these categories, the strongest predictor appeared to be Helplessness and Passiveness in the face of problems. The method of suicide attempt is a variable related to psychosocial determinants of suicidal behaviour. The findings of this study may prove useful in the design and implementation of therapeutic activities focused on persons who attempted suicide. Recognising the particular manifestations of indirect self-destructive behaviours of an attempter can guide implementation of therapeutic measures, for him/her e.g. via strengthening coping skills and eliminating risk factors for self-harm.

## Introduction

The choice of a suicide attempt method is an important issue because it provides information about the motivation, psychological functioning, strivings, and aims of the attempter. It may be an indication of certain traits or even mechanisms governing his/her psychology. Such suicide method characteristics as the degree of body integrity breach or violence not only underlie the choice of the method, but also reveal information about the personality of the attempter. Therefore, it can help develop the best therapy and prevention programme for him/her.

The suicide literature includes a number of studies devoted to the methods chosen in both completed and attempted suicides. Less attention is paid to the factors directly or indirectly associated with them. Suicidal acts are regarded as the signs of self-aggression or self-destructiveness, mainly direct self-destructiveness. Direct self-destructiveness is a concept very close to what Silverman et al. [1, 2] termed the “Risk-Taking Thoughts and Behaviors with Immediate Risk” (A.1), which may result in no injury or in injury or in death, and “Suicide-Related Behaviors” (B.3) with some degree of suicidal intent (suicide attempt), especially with fatal outcome (suicide). There are only a few papers dealing with suicides or suicide attempts as the manifestations of indirect self-destructiveness.

This issue is of vital importance since indirect self-destructiveness may lead to suicide attempts or death by suicide [3, 4].

The psychological mechanisms underlying the choice of a suicide method are still unknown [5]. The method of suicidal behaviour is associated with motivational processes [6, 7] and the psycho(patho)logical mechanisms and traits and of an individual.

Indirect self-destructiveness may be close in meaning to the “Risk-Taking Thoughts and Behaviors with Remote Risk” (A.2), which may result in no injury or in injury or in death [1, 2], e.g. smoking and sexual promiscuity.

Chronic self-destructiveness is defined as behaviours involving the generalised tendency to engage in acts that increase the probability of experiencing future negative consequences and/or reduce the probability of attaining future positive ones. The individuals who are primarily motivated by current emotional factors are more likely to engage in self-destructive acts than the persons motivated by more distant cognitive considerations. Moreover, individuals high in chronic self-destructiveness, compared to those with low scores, are more likely to be in treatment for drug or alcohol abuse, to report having cheated in courses, to have had traffic violations, to report having gone through a rebellion stage in adolescence, and to postpone obtaining a medical test for cancer [8]. Indirect (chronic) self-destructiveness is also considered the behaviours whose probable negative consequences are mediated by additional factors, and there is a probability of a relationship between behaviour and injury. Thus defined indirect/chronic self-destructiveness includes not only the undertaking of, but also abandoning (commission or omission) of action; this is related to exposure to dangerous situations, or to neglecting one’s health and safety. While acute/direct self-destructive behaviour involves a conscious and wilful intent to injure oneself, sometimes with a suicidal intent, the chronic/indirect self-destructiveness refers to actions and situations extended over a period of time, where an individual is unaware of or disregards their long-term adverse effects [4, 9, 10]. Five categories of indirect self-destructiveness can be distinguished. Transgression and Risk includes violation of social norms, e.g. school regulations or the principles and norms of social coexistence as well as risky behaviours undertaken for momentary pleasure, e.g. reckless driving, mainly to show off, and gambling. Within this category, one can also find yielding to temptations, impulsiveness, and seeking excitement in hazardous activities. Poor health maintenance includes behaviours hazardous to health, e.g. excessive eating and drinking, neglecting medical check-ups or ignoring doctor’s recommendations. Personal and social neglects refer to neglecting one’s duties or matters important to an individual both in private terms and in social relations. Lack of planfulness means acting without any previous schedule or a future perspective. Helplessnes and passiveness includes abandonment of action under conditions when such action could stop one’s suffering or prevent hazard [8–10].

Indirect self-destructiveness is a form of harming oneself that distinctly differs from direct self-destructiveness or self-aggression.

As mentioned above there are only a few papers dealing with the relationships between suicides or suicide attempts and indirect self-destructiveness [*cf* 3, 4].

The aim of the present study was to examine the relationship between individual manifestations of indirect self-destructiveness and the methods of suicide attempt as well as to explore the indirect self-destructive behaviours predictive of particular suicide methods.

## Methods

### Participants

The study was conducted among persons who attempted suicide using different methods and were admitted to hospital thereafter. The examinations were carried out after the patients had completed their medical treatment. The study population comprised 147 participants (114 females and 33 males) between 23 and 33 years of age.

When the treatment of the somatic effects of suicide attempt had been completed, the patients underwent standard psychiatric and psychological examinations (mostly at mental health centres) conducted by psychiatrists and clinical psychologists. The findings revealed no psychotic disorders or mental retardation among the study population. Then the CS-DS questionnaire was administered to the subjects by well-trained and experienced clinical psychologists. The entire research project lasted approximately 12 months.

The examination was anonymous and the participation was voluntary. It is worth noting that there were no refusals. Informed consent, according to the Helsinki Declaration recommendations, was obtained from each participant and the health centre management granted permission to perform the study on their patients.

### Materials

In order to examine indirect self-destructiveness and its manifestations, the Polish version of “Chronic Self-Destructiveness Scale” by Kelley (CS-DS), as adapted by Suchańska, was administered. Kelley’s scale assessing chronic (indirect) self-destructiveness as a generalised tendency includes four categories of behaviour: carelessness, poor health maintenance, evidence of transgression, and lack of planfulness. The ultimate version consists of an internal, coherent set of 52 items [8].

Both the original scale and its Polish adaptation are characterised by high reliability and validity. For the original scale, the reliability (internal consistency, Cronbach’s alpha) ranged from 0.90 to 0.98 [8]. In the Polish adaptation, the values varied from 0.799 to 0.811 for reliability (Cronbach’s alpha) and from 0.752 to 0.861 for content validity [10].

The Polish scale comprises the following categories: Transgression and Risk (A1; e.g. I use or have used street drugs; An occasional fight makes a guy more of a man), Poor Health Maintenance (A2; e.g. I have a complete physical examination once a year; I always do what my doctor or dentist recommends), Personal and Social Neglects (A3; e.g. I usually meet deadlines with no trouble; I am frequently late for important things), Lack of Planfulness (A4; e.g. I just don’t know where my money goes; I hate any kind of schedule or routine), and Helplessness and Passiveness in the face of problems (A5; e.g. Sometimes I don’t seem to care what happens to me; It’s easy to get a raw deal from life), the scores for which sum up to one global score for indirect self-destructiveness [9, 10].

The correlation and regression analyses were used to examine the relationship between the different manifestations of indirect self-destructiveness and the particular methods of suicide attempt, as well as to explore the indirect self-destructive behaviours as predictive of a particular suicide method. The quantitative data were analysed using the mean, standard deviation, correlation coefficient (Kendall Tau) and stepwise multiple regression analysis; *p* ≤ 0.05 was considered significant. All the calculations were made using *Statistica PL 8.0 for Windows* [11].

## Results

The participants used several methods in their suicide attempts, these including drug overdose, wrist cutting, hanging, jumping from a height, asphyxia, self-poisoning and walking into traffic (Table 1).

**Table 1:** Suicide attempt methods

Rank	Suicide attempt method	*n*	%
1.	Drug overdose	99	41.60
2.	Wrist cutting	60	25.21
3.	Hanging	39	16.39
4.	Jumping from a height	21	8.82
5.	Asphyxia	9	3.78
6.	Poisoning	5	2.10
7.	Walking into traffic	5	2.10
Total		238	100.00

The most prevalent suicide method was drug overdose (41.60 %) and wrist cutting (25.21 %), while the least frequent were self-poisoning and walking into traffic (2.10 %). The predominant suicide method differed considerably from that found in other studies (e.g. hanging or poisoning) [6, 7, 12, 13]. In the present study, the use of firearms in a suicide attempt was not reported [*cf* 4]. The findings also revealed that for as many as 93 participants, the suicide attempt was a repeated event. The prevailing methods of reccurrent attempt were drug overdose and wrist cutting, and the least frequent were asphyxia and walking into traffic. Among the participants who repeated suicide attempt(s), no-one attempted poisoning.

The scores for the study population in the CS-DS scales are presented in Table 2.

**Table 2:** CS-DS scores for the study population

Variable	Mean	SD
Indirect self-destructiveness	154.041	22.762
Transgression (A1)	53.551	11.305
Poor health maintenance (A2)	31.306	7.253
Personal and social neglects (A3)	33.429	6.723
Lack of planfulness (A4)	21.898	5.456
Helplessness and passiveness (A5)	10.020	1.981

The general scores for CS-DS and its individual categories significantly correlated (Kendall Tau) with particular methods of suicide attempt (Table 3). Indirect self-destructiveness as a generalised tendency was significantly related with drug overdose (*p* = 0.00001), poisoning (*p* = 0.01), and walking into traffic (*p* = 0.04) [*cf* 4]. Transgression scores significantly and positively correlated with drug overdose (*p* = 0.0001) and wrist cutting (*p* = 0.05), but negatively with poisoning (*p* = 0.01). The scores for Poor Health Maintenance significantly and positively correlated with drug overdose (*p* = 0.0000) and poisoning (*p* = 0.03), but negatively with asphyxia (*p* = 0.00009). A significant positive correlation was found between the scores for Personal and Social Neglects and poisoning (*p* = 0.008) or walking into traffic (*p* = 0.0006). The scores for Lack of Planfulness significantly correlated with asphyxia (*p* = 0.001). There were significant positive correlations between Helplessness and Passiveness and drug overdose, wrist cutting, jumping from a height, poisoning, and walking into traffic. A negative correlation with hanging (*p* = 0.006) was noted. The risk of repeated suicide attempt was found to correlate with indirect self-destructiveness as a generalised tendency (*p* = 0.007) [*cf* 4] as well as with Transgression (*p* = 0.000006).

**Table 3:** Correlations (Kendall Tau) between manifestations of indirect self-destructiveness and suicide attempt methods

Variable	Drug overdose	Wrist cutting	Hanging	Jumping from a height	Asphyxia	Poisoning	Walking into traffic	Recurrence
Indirect self-destructiveness	0.366					0.667	0.542	0.217
	*p* = 0.00001	ns.	ns.	ns.	ns.	*p* = 0.01	*p* = 0.04	*p* = 0.007
Transgression (A1)	0.318	0.162				−0.660		0.367
	*p* = 0.0001	*p* = 0.05	ns.	ns.	ns.	*p* = 0.01	ns.	*p* = 0.00006
Poor health maintenance (A2)	0.590				−0.662	0.583		
	*p* < 0.0000	ns.	ns.	ns.	*p* = 0.00009	*p* = 0.03	ns.	ns.
Personal and social neglects (A3)						0.739	0.917	
	ns.	ns.	ns.	ns.	ns.	*p* = 0.008	*p* = 0.0006	ns.
Lack of planfulness (A4)						0.870		
	ns.	ns.	ns.	ns.	ns.	*p* = 0.001	ns.	ns.
Helplessness and passiveness (A5)	0.338	0.297	−0.261	0.377		0.956	0.857	
	*p* = 0.0001	*p* = 0.0005	*p* = 0.006	*p* = 0.002	ns.	*p* < 0.0000	*p* = 0.003	ns.

However, the correlations themselves are not informative enough, and we were wondering what is the set of variables related to indirect self-destructiveness that best explains the use of a particular suicide method by the attempter. To this end, a stepwise multiple regression analysis was used (Table 4). All the categories of indirect self-destructive behaviour were included in the initial model of regression equation.

**Table 4:** Stepwise multiple regression for manifestations of indirect self-destructiveness and suicide methods

Dependent variable: drug overdose				
Multiple regression coefficient *R* = 0.527				
Significance of regression equation *F*(5, 141) = 10.830, *p* < 0.00000				
Variables	Beta (β)	Std. B (β)	*t*(141)	*p*-level
A1—Transgression	0.134	0.005	1.658	ns.
A2—Poor health maintenance	0.492	0.032	6.115	*p* < 0.00000
A3—Personal and social neglects	−0.055	−0.004	−0.657	ns.
A4—Lack of planfulness	−0.095	−0.008	−1.119	ns.
A5—Helplessness, passiveness	0.048	0.011	0.626	ns.

Dependent variable: wrist cutting				
Multiple regression coefficient *R* = 0.365				
Significance of regression equation *F*(5, 141) = 4.329, *p* < 0.001				
Variables	Beta (β)	Std. B (β)	*t*(141)	*p*-level
A1—Transgression	0.254	0.011	2.866	0.004
A2—Poor health maintenance	−0.098	−0.006	−1.119	ns.
A3—Personal and social neglects	−0.080	−0.005	−0.884	ns.
A4—Lack of planfulness	−0.201	−0.018	−2.160	0.03
A5—Helplessness, passiveness	0.273	0.068	3.262	0.001

Dependent variable: jumping from a height				
Multiple regression coefficient *R* = 0.254				
Significance regression equation *F*(5, 141) = 1.939, *p* < 0.09				
Variables	Beta (β)	Std. B (β)	*t*(141)	*p*-level
A1—Transgression	−0.060	−0.001	−0.652	ns.
A2—Poor health maintenance	−0.013	−0.000	−0.137	ns.
A3—Personal and social neglects	−0.207	−0.011	−2.183	0.03
A4—Lack of planfulness	0.188	0.012	1.943	ns.
A5—Helplessness, passiveness	0.187	0.033	2.150	0.03

Dependent variable: asphyxia				
Multiple regression coefficient *R* = 0.331				
Significance of regression equation *F*(5, 141) = 3.462, *p* < 0.005				
Variables	Beta (β)	Std. B (β)	*t*(141)	*p*-level
A1—Transgression	−0.082	−0.002	−0.918	ns.
A2—Poor health maintenance	−0.327	−0.011	−3.665	0.0003
A3—Personal and social neglects	0.080	0.003	0.871	ns.
A4—Lack of planfulness	0.075	0.003	0.804	ns.
A5—Helplessness, passiveness	0.099	0.012	1.178	ns.

Dependent variable: poisoning				
Multiple regression coefficient *R* = 0.452				
Significance of regression equation *F*(5, 141) = 7,234, *p* < 0.0000				
Variables	Beta (β)	Std. B (β)	*t*(141)	*p*-level
A1—Transgression	−0.277	−0.003	−3.256	0.001
A2—Poor health maintenance	0.040	0.000	0.482	ns.
A3—Personal and social neglects	−0.023	−0.000	−0.274	ns.
A4—Lack of planfulness	0.215	0.006	2.414	0.01
A5—Helplessness, passiveness	0.361	0.026	4.506	0.00001

Dependent variable: walking into traffic				
Multiple regression coefficient *R* = 0.282				
Significance of regression equation *F*(5, 141) = 2.443, *p* < 0.03				
Variables	Beta (β)	Std. B (β)	*t*(141)	*p*-level
A1—Transgression	0.012	0.000	0.142	ns.
A2—Poor health maintenance	−0.154	−0.003	−1.700	ns.
A3—Personal and social neglects	0.256	0.005	2.712	0.007
A4—Lack of planfulness	−0.031	−0.000	−0.334	ns.
A5—Helplessness, passiveness	0.111	0.008	1.292	ns.

Dependent variable: repeated suicide attempt (recurrence)				
Multiple regression coefficient *R* = 0.301				
Significance of regression equation *F*(5, 141) = 2.819, *p* < 0.01				
Variables	Beta (β)	Std. B (β)	*t*(141)	*p*-level
A1—Transgression	0.333	0.014	3.668	0.0003
A2—Poor health maintenance	−0.029	−0.002	−0.325	ns.
A3—Personal and social neglects	−0.015	−0.001	−0.164	ns.
A4—Lack of planfulness	−0.086	−0.008	−0.910	ns.
A5—Helplessness, passiveness	−0.008	−0.002	−0.03	ns.

As shown in Table 4, regression analysis for drug overdose revealed that Poor Health Maintenance (*p* = 0.0000) was a significant predictor, which is somewhat paradoxical since pharmaceuticals are supposed to support or restore health. For wrist cutting, three categories of indirect self-destructive behaviour were significant predictors: Transgression (*p* = 0.004), Lack of Planfulness (*p* = 0.03), and Helplessness and Passiveness (*p* = 0.001). No significant predictive variables were found for hanging, the suicide method ranked third by frequency. There were two significant predictors for jumping from a height: Personal and Social Neglects (*p* = 0.03) and Helplessness and Passiveness (*p* = 0.03). For asphyxia, only Poor Health Maintenance was a significant predictor (*p* = 0.0003). With regard to poisoning, three categories of indirect self-destructiveness were significant: Transgression (*p* = 0.001), Lack of Planfulness (*p* = 0.01), and Helplessness and Passiveness (*p* = 0.00001). Only one statistically significant predictor, namely Personal and Social Neglects (*p* = 0.007), was found for walking into traffic, the least frequent suicide method. An important predictive variable for repeated suicide attempt proved to be Transgression (*p* = 0.0003).

As shown in Tables 3 and 4, there were differences between the results of correlation and regression analyses for relationships between individual categories of indirect self-destructiveness and suicide methods. In particular, significant correlations were found (e.g. between drug overdose and Transgression) that were not confirmed in the regression equation, and conversely, the regression analysis revealed significant predictors (e.g. Lack of Planfulness for wrist cutting) that did not occur in the correlations.

The largest number of associations in the correlation and regression analyses were found for self-poisoning, whereas the fewest were found for jumping from a height.

## Discussion

The findings of the present study cannot be compared with other results since there were practically no studies that would address the relationship between the particular categories of indirect self-destructiveness and the methods of suicide attempt. The projects conducted thus far concerned only the associations between what is now termed “indirect self-destructiveness” and suicides. In the present study, we investigated the predictors that have not as yet been given enough consideration. Accordingly, we have focused on the relations between particular categories of indirect self-destructiveness and the methods of suicide attempt.

As revealed by the study results, Helplessness and Passiveness in the face of problems correlated with all the suicide methods. This finding is not surprising if the suicide attempt is perceived as an expression of the attempter’s hopelessness and helplessness in a difficult life situation. No matter whether the true intention of the attempter was to definitively end his/her life, or whether it was a desperate “crying for help”, the act of suicide seemed to be the best solution. This is consistent with the viewpoints expressed by other authors—suicide has been perceived as a final solution to obtain release or escape (often from psychological pain) [2].

Among the methods of suicide attempt, the strongest correlation (0.667) was found between poisoning (a relatively infrequent method) and indirect self-destructiveness as a generalised tendency, and poisoning and all the categories of indirect self-destructiveness, i.e. Helplessness and Passiveness, Lack of Planfulness, Personal and Social Neglects, Poor Health Maintenance and Transgression (negative coefficient). Given these data, one can assume that the persons who attempted poisoning exhibited the strongest self-destructive tendencies, i.e. a desire to definitively end their lives. This is concordant with an observation that among the patients who poisoned themselves, highly lethal attempts were related to a higher risk for death by suicide [13]. However, it is worth noting that this suicide method is characterised by the lowest degree of body integrity breach and hence the lowest level of self-aggressiveness [6, 7], which implies that self-aggressiveness should not be regarded as a counterpart of indirect self-destructiveness. The data from correlation and regression analyses indicate that the persons who tried self-poisoning chose a method that would let them die quietly and peacefully, without any spectacular effects that are characteristic e.g. of wrist cutting, jumping from a height, or walking into traffic (negative Beta and negative correlation with Transgression). The Lack of Planfulness among these persons is reflected in their acting in despair and choosing “what was at hand”, e.g. toxic household chemicals, as the agent of poisoning. The purport of Poor Health Maintenance is clear: it is difficult to speak about health care in the case of self-poisoning. A similar observation refers to Personal and Social Neglects: suicide attempt is an expression of extreme neglect with respect to everything that is important to the individual in the personal and social dimension.

The most prevalent method of suicide attempt was drug overdose. Statistical analysis of data for participants who attempted drug overdose revealed interesting findings. The medications whose purpose is to support or restore health are paradoxically connected with neglecting one’s health. Therefore, a sudden or unusual interest in pharmaceuticals, which is expressed by someone who usually neglects his/her health, should be a warning signal.

Among the prevailing suicide methods is wrist cutting. In this case, Transgression may be reflected by theatricality and spectacular effects, which are intended to evoke strong emotions among the witnesses of the attempt, e.g. family, rescue team, etc. Wrist cutting is often planned (negative Beta for Lack of Planfulness) and, therefore, belongs to the less lethal methods. Other reports present similar conclusions: cutting was not associated with higher suicide mortality rate; this method usually represents low suicide intent and rather reflects poor emotional regulation [13, 14].

Regression analysis revealed no significant predictors for hanging as the suicide method, but a negative correlation with Helplessness was found. Bearing in mind that helplessness limits definite actions, the purport of this result is as follows: compared with other methods, hanging requires greater psychological and physical effort on the part of the attempter. There are a number of choices to be made, decisions to take and activities to carry out, including such “technical” issues as how to fasten up the rope, put the loop around the neck, etc.

In the case of jumping from a height, which is one of the most lethal methods of suicide, helplessness may also be reflected in the fact that the attempter does not have a direct impact on what happens after the jump. This may be perceived as a kind of symbolism: “come what may” which means a total resignation from life. Negative Beta for Personal and Social Neglects may indicate a “care” at least in this case: the attempter chose a method, which is most likely to produce fatal effects. Besides, the method is characterised by a high level of self-aggressiveness and body integrity breach: it causes numerous external and internal injuries. Therefore, one can see that self-aggressiveness is not the same as indirect self-destructiveness; jumping from a height is not significantly related to indirect self-destructiveness as a generalised tendency.

With regard to asphyxia, Poor Health Maintenance turned out to be a significant predictor (negative correlation and Beta). These associations may be related to the lowest degree of body integrity breach; compared with other methods, asphyxia causes only minor external and internal changes.

Personal and Social Neglects proved to be important predictors for walking into traffic which is the rarest of suicide methods. Personal neglect is clear in this case. As for the social neglects, an attempter engages other people (driver, passengers, passers-by) in his/her problems. For the possible witnesses, and most of all for the driver, such a suicide act is a strong and unpleasant experience. The driver of a car is at risk not only for psychological by trauma, but also may be injured by the impact the attempter or an accident from swerving to avoid hitting him/her. Apart from that, the driver may suffer from a feeling of guilt for the attempter’s death or injuries, or he/she may at least have a sense of perpetration or complicity.

Literature reports support the presumption that suicide attempt is a risk factor for death by suicide. Moreover, the history of a prior suicide attempt is a statistically significant risk factor associated with future self-destructive behaviours including death [1, 4, 15, 16]. The risk of repeated suicide attempt is strongly related to Transgression. The role of the egocentric attitude as well as the lack of respect for the principles should be underlined. An issue worth considering is the sense of helplessness and hopelessness that can lead to depression and suicide attempts [17, 18]. Many persons who attempted suicide were also found to have impulse control problems [19] and a high level of aggression and impulsivity [20].

Fortunately, the persons attempting suicide may later on change their attitude to life and problem coping, as well as their self-assessment; patients who survive a suicide attempt may eventually develop more effective coping, enhanced self-esteem, and a more optimistic attitude toward life. Worthy of mention is a 10-year follow-up study of adolescents who had attempted suicide: the findings revealed that as much as 70.50 % of respondents stated that they were happy [21]. Since neither the suicide attempt itself nor the suicide method precludes a possibility of leading a happy life afterwards, it is essential to offer the attempters any possible psychological guidance and to mobilise them to take the chance [*cf* 4].

## Conclusions

The findings of our study indicate that many indirect self-destructive behaviours may be a warning signal of an intended suicide and they may even be predictive of a particular suicide method to be used by the attempter. As such, they may prove useful in the design and implementation of the therapeutic activities intended for persons after suicide attempts (attempters or survivors) as well as in comprehensive preventive activities and psychoprophylaxis. Therefore, it can help develop the best therapy and prevention programme for the attempter. Knowing the method the patient used in suicide attempt may be of great help for the therapist, since it is a variable related to the psychosocial determinants of suicidal behaviour. The basic activities in psychotherapy for suicide survivors should consist in strengthening the psychological resources of an individual, mainly his/her abilities to cope with problems and difficult life situations, as well as eliminating the risk factors for self-harm. It is equally important to work with the attempters on developing interpersonal skills and social relations. This refers particularly to persons who attempted suicide by walking into traffic or those who tried self-poisoning. The control of emotions and impulses, and respect for principles and borderlines should be considered when working with patients with medication abuse, tried wrist cutting or repeated suicide attempts. Moreover, the optimistic reframing of negative life events may have implications for general prevention of suicidal activity [22], since pessimism is a well recognised suicide risk factor [4, 17, 18].

The present study does not cover all the aspects of this complex and profound problem and will require further research, particularly in the context of the psychological and therapeutic work with suicide attempters and survivors.

*Limitations*: In the present study, we have confined to the predictors that have not been given enough consideration thus far. Accordingly, we have focused on the relations between the particular categories of indirect self-destructiveness, rather than a generalised tendency, and the methods of suicide attempts. More detailed aspects of this complex problem could be addressed in further research.

In addition, the gender differentiation in relationships between indirect self-destructiveness, its manifestations and methods of suicide attempts seems to be an interesting issue for further studies.
